# HIV disclosure to sexual partner and associated factors among women attending ART clinic at Mekelle hospital, Northern Ethiopia

**DOI:** 10.1186/1471-2458-14-746

**Published:** 2014-07-23

**Authors:** Mussie Alemayehu, Alemseged Aregay, Abrhet Kalayu, Henock Yebyo

**Affiliations:** Department of Public Health, Mekelle University College of Health Sciences, Mekelle, Ethiopia; Department of Nursing, Mekelle University College of Health Sciences, Mekelle, Ethiopia; Department of Nursing, Sheba University College, Mekelle, Ethiopia

**Keywords:** Disclosure, HIV, Women, Mekelle, Tigray, Ethiopia

## Abstract

**Background:**

Disclosure of HIV positive status has two sets of contrary effects. It may motivate partner for Voluntary Counseling and Testing; on the other hand, it may cause blame, discrimination, depression and loss of economic support. Consequently, HIV positive status disclosure among women has become one of the major concerns that should be addressed in HIV prevention and control activities. This study aimed at assessing the magnitude and factors related to HIV positive status disclosure to sexual partners among HIV positive women.

**Methods:**

A cross sectional study was conducted in Mekelle hospital from July 10–26, 2013 to collect data from 315 HIV positive women using a systematic random sampling. Descriptive and multiple logistic regression analyses were performed using SPSS 20 for windows to estimate indicators and effect sizes of the predictors on HIV disclosure status to partners.

**Results:**

The proportion of HIV disclosure status to their partner was 63.8%. Women who knew the HIV status of their sexual partner and those who got pretest counseling had a positive association with HIV disclosure with AOR of 16.9 (95% CI: 8.11, 35.21) and AOR of 2.8 (95% CI: 1.83, 4.28). Mothers with two years or beyond, since they knew their HIV status had more odds (AOR = 3. 2, 95% CI: 1.7, 6.29) to disclose their HIV status to their partner. Mothers who had seen people with HIV positive who disclose their HIV status to the community (AOR = 2.1, 95% CI: 1.08, 4.01) and those who had a discussion prior to HIV testing (AOR = 4.87, 95% CI: 2.45, 9.71) were more likely to disclose their HIV status than their counterparts.

**Conclusions:**

The rate of HIV disclosure to their partner was low. Knowledge of HIV status of partner, receiving pretest counseling, longer time since the HIV testing, know people who disclose their status to the community and having discussion prior to HIV testing could influence disclosure of HIV status of mothers to their partners.

## Background

The Human Immunodeficiency Virus (HIV) epidemic continues to be a major public health problem worldwide. At the end of 2010, there were an estimated 34 million people living with HIV/AIDS. Sub-Saharan Africa (SSA) bears an inordinate share of the global HIV burden accounting for 68% of all patients living with HIV/AIDS (PLWHA); of which, 59% of them were women [1, 2]. Though the HIV prevalence in Ethiopia is lower than many SSA countries, its epidemic led it to be one of the countries with huge magnitude HIV-infected people worldwide.

According to the 2010 single point estimate, the estimated adult HIV/AIDS prevalence in Ethiopia was 2.4% [3]. A larger proportion of these are under treatment. A number of factors determine for the success of the treatment. HIV positive status disclosure among women has become one of the major concerns that should be addressed in HIV prevention & control activities which is one of the factors that determine the success of HIV/AIDS related programs [4]. Moreover, it may affect the rate of transmission of the virus to their sexual partners [5, 6]. Early disclosure of HIV positive status of women to sexual partner is crucial and helpful for treatment adherence, HIV transmission reduction, PMTCT interventions, partner testing and acceptance of referrals for HIV-related care, treatment and support.

Nevertheless, disclosure of HIV positive status has two sets of contrary effects. It may motivate partner for Voluntary Counseling and Testing (VCT), reduce risk behaviors, and increase acquisition of support and adherence to ART [7, 8]. On the other hand, it may cause blame, discrimination, abandonment, depression and loss of economic support and disruption of family relationship. For the fear of these risks, patients may not disclose their HIV positive status [9, 10] which, in turn, may have a negative effect on women’s treatment outcome of HIV/AIDS care. However, evidences are scarce regarding to the HIV discloses and factors determining HIV disclose to their partners.

This study aimed to assess the level of HIV disclosure and factors related to the disclosure among women living with HIV in Mekelle hospital, northern Ethiopia.

## Methods

An institutional based cross-sectional study was conducted in Mekelle hospital from July 10–26,2013. The total population of the area is 233,000, with 119,800 females and the health service coverage of Mekelle city was 90% [11]. Tigray is the dominant ethnic group in Mekelle. All HIV positive women aged 18 years or above who have been registered and getting clinical service at the ART clinic in Mekelle hospital were considered as source population. Clients who were critically ill during the data collection period were excluded from the study.

To determine the sample size, a single population proportion formula with a proportion of disclosure in Mettu and Gore town was 69% [5], a confidence level of 95%, and a 5% degree of precision were used. With assumption of 10% non response rate, the total sample size computed was 315.

Systematic random sampling was used to select the study subjects. There were a total of 2,218 female patients who follow chronic ART care in the hospital. Based on the sample fraction, women were selected at equal interval using systematic random sampling. Those who didn’t fulfil the inclusion criteria were excluded and the next women fulfilling the criteria were included. However, women who refused to participate were excluded from the study without replacement.

Structured and pre-tested questionnaire, guided by the interviewer, was used to collect the information. It was first prepared in English and then translated into the local language (Tigrigna) and then translated back to English to check for consistency of the questions. Information collected included socio demographic and economic characteristics, individual and partner factor, health and health related factors and disclosure of HIV status. The questionnaire was adapted from different literatures and considering the local situation of the study subjects [5, 12, 13]. Four diploma clinical nurses who can speak the local languages were employed in the data collection process. Two BSc nurses were posted for supervision. Training was given to the data collectors and supervisors for two consecutive days on the purpose of the study, the contents of the questionnaire, particularly on issues related to the confidentiality of the responses and the rights of respondents. One week prior to the data collection, the questionnaire was pre-tested at Quiha district in 16 of women.

The data collected was entered into and analyzed using SPSS 20 for windows (SPSS Inc. Version 20., Chicago, Illinois). Exploratory data was run to check for missing values, outliers and expected values of the categories for regression. Descriptive and multiple logistic regression was used to estimate the respective indicators, and effects of factors on the HIV disclosure of the mothers to their partners. Collinearity among independent factors was checked using VIF. The sample effect size was estimated using OR and the parameters were estimated using 95% confidence interval of the OR. For all the analyses, P-value less than 0.2 was considered statistically significant.

The study was approved by the ethical committee of Mekelle University, College of health science research and community service committee. Formal letter of permission was obtained from Mekelle University and Tigray Regional Health Bureau. A letter was also obtained from the director of Mekelle hospital that assured to continue the study. Verbal consent was obtained from the study respondents individually. The right of the respondent to withdraw from the interview or not to participate at all was assured.

## Results

### Socio demographic and economic characteristics

A total of 315 HIV positive women had participated in the study with a response rate of 100%. The majority of the respondents were urban 259 (82%) and Orthodox Christianity followers 276 (84.8%). One hundred thirty nine (44.1%) and 220 (69.8%) of the respondents were married and literate, respectively. Pertaining to the educational status of their partner, 97 (30.8%) attended college or university. The overall respondents age ranges from 20 to 65 with mean of age 34.9 (±8.1) years (Table 1).

**Table 1:** **Socio-demographic characteristics of the women on ART at Mekelle Hospital, Northern Ethiopia**

Variable	Number	Percent
Residence		
Urban	259	82.2
Rural	56	17.8
Ethnicity		
Tigray	293	93
Amhara	22	7
Age		
20-24	22	7
25-29	57	18.1
30-34	76	24.1
35-39	68	21.6
40-44	53	16.8
> = 45	39	12.4
Occupation		
Daily worker	35	11
Governmental employee	57	18.1
NGO employee	50	15.9
Housewife	102	32.4
Merchant	15	4.8
Farmer	56	17.8
Marital status		
Single	25	7.9
Married	139	44.1
Divorced	72	22.9
Separated	10	3.2
Widowed	69	21.9
Religion		
Orthodox	267	84.8
Muslim	36	11.4
Others*	12	3.8
Educational status of the respondents		
Illiterate	95	30.2
Write and read	16	5.1
Primary school (1–8)	101	32.1
Secondary school (9–12)	41	13
College or university level	62	19.7
Educational status of their partner		
Illiterate	37	11.7
Write and read	36	11.4
Primary school (1–8)	79	25.1
Secondary school (9–12)	66	21.0
College or university level	97	30.8

*Others (catholic and protestant).

### Health and health care related factors of the respondents

The length of time since the mothers knew their HIV status differed. In nearly 60% of them, it has been 2 years or beyond, since they knew their HIV status. Five out of ten mothers were tested their HIV status in VCT while the rest were tested in different situations. In 169 (53.5%) of the mothers, illness caused them to get tested for an HIV test. Sixty percent of the mothers didn’t have a discussion about HIV test with their partners. During HIV testing, only 28 (11.5%) came with their partners and were counseled together. The rest of the women came alone.

More than half, 175 (55.6%), of the women had been staying in ART and/or Pre-ART for 2 years or over. Nearly 84% of the women were under the ART during the study period. With regard to their perception, 253 (80.3%) thought ART initiation is important for disclosure of HIV status. One hundred fifty seven (85%) of the HIV positive women were living in the same house with their current sexual partner for at least one year (Table 2).

**Table 2:** **Health and health care related factors of respondents at Mekelle Hospital, Northern Ethiopia**

Variables	Number	Percent
Time to know your HIV sero positive		
<=2 years	124	39.4
>2 years	191	60.6
Place of test for HIV		
ANC	78	24.8
Delivery	24	7.6
VCT	157	49.8
Providing is initiating counseling and testing	56	17.8
Discussion before test with partner		
Yes	99	31.4
No	216	68.6
Relationship before test with partner		
Smooth	218	69.2
Disagreement	97	30.8
Initiating factor to test for HIV		
Becoming sick	169	53.5
Others*	146	46.5
Duration of follow up in ART or pre ART service		
<=2 years	140	44.4
>2 years	175	55.6
History of Hospital admission in the last 12 months		
Yes	60	19.0
No	255	81.0
Current category of respondents		
Pre-ART Rx	45	14.3
ART Rx	270	85.7
Initiating ART is important for disclosure		
Yes	253	80.3
No	62	19.7
Regular sexual partner in the last 12 months (n = 315)		
No	130	41.3
Yes	185	57.7
Live in the same house with regular sexual partner (n = 185)		
Yes	157	85
No	28	15
Numbers of years lived with sexual partner (n = 157)		
<=1 year	23	14.6
>1 year	134	85.4

*Friends, family and health professionals.

### Disclosure status, sexual experience and individual factors of the respondents

Nearly 64% of the HIV positive women disclosed their HIV status to their partners. Of those, 169 (84%) of the respondents disclosed their status to their usual partner, whereas 32 (16%) to their causal partner. With regard to intention, 66.7% of the undisclosed women didn’t have an intention to disclose their HIV status to their partner. Majority of the women (85%) disclosed their status after ART initiation. The main reasons mentioned by the women to disclose their status were the influence in due by another HIV positive individual (31.8%) and seeking support from their partner (26.4%). Whereas, on the contrary, the main reason for not disclosing their status was fear of separation (22.8%). Over half of the women (53%) haven’t recognized any person who discloses his/her HIV status to the community.

Pertaining to their sexual practice, 55.2% of the women practiced sexual intercourse in the last six months of the interview. Of these, 64.6% had always used condoms (Table 3).

**Table 3:** **Sexual experiences and factors related to HIV disclosure among women at Mekelle Hospital, Northern Ethiopia**

Variables	Number	Percent
Period of disclosure to their partner after testing HIV (n = 201)		
Immediately	26	13
<1 months	36	18
1-6 months	52	26
>6 months	87	43
Type of partner for disclosure (n = 201)		
Steady sexual partner	169	84.0
Causal sexual partners	32	16.0
Time of disclosure to sexual partner (n = 201)		
Before starting ART	30	15
After initiating ART	171	85
Reasons for decide to discloser (n = 201)		
Peer support	10	5.0
Mass media	55	27.4
HIV positive individuals	64	31.8
Self	48	23.9
Partner	24	11.9
Reason for their disclosure (n = 201)		
To get support from their sexual partner	53	26.4
To decrease HIV transmission	49	24.4
To benefit sexual partner to get medical care	42	20.9
To adhere ARV drug regimen	32	15.9
The presence of ARV drug at home	14	7
Spiritual responsibility	11	5.4
Reasons for non disclosure (n = 114)		
Fear of abandonment	26	22.8
Fear of a shaming family	9	7.9
Lack of communication skill	9	7.9
Loss of confidentiality	8	7
Fear of hurt	14	12.3
Fear of accusation of infidelity	10	8.8
Fear of stigma and discrimination	14	12.3
Not enough time to discuss	21	18.4
I do not want him to worry	3	2.6
Time of intention for disclosure (n = 38)		
Within a week	16	42.1
Within a month	22	57.9
Have you had sex in the last 6 months (n = 315)		
Yes	174	55.2
No	141	44.8
Use condom (n = 174)		
Yes	116	66.7
No	58	33.3
Time of using condom (n = 116)		
Always	75	64.6
Some times	41	35.4

### Factors associated with HIV disclosure

The result of the multivariate analyses showed that women who knew their partner’s HIV status were more likely to disclose their own (AOR = 16.9; 95% CI: 8.11, 35.21). Pretest counseling was also positively associated with HIV disclosure of the women as compared to their counterparts (AOR = 2.8, 95% CI: 1.83, 4.28). Women who knew their HIV status for longer than 2 years had 3 times higher odds of disclosing their HIV status to their partner than women with relatively shorter (AOR = 3.2, 95% CI (1.7, 6.29). The result substantiates the help of model HIV positive individuals in disclosing HIV status. Women who had seen an HIV positive person disclosing his/her status to the community had 2 times higher odds to disclose their HIV status (AOR = 2.1, 95% CI: 1.08, 4.01). Discussion with their partners on the need of HIV testing was associated with higher odds of disclosing women’s HIV status to their partner (AOR = 4.87, 95% CI: 2.45, 9.71) (Table 4).

**Table 4:** **Predictors of HIV disclosure among women on ART at Mekelle Hospital, Northern Ethiopia**

Characteristics	Disclosure of HIV status		COR	AOR
	Yes	No		
	No (%)	No (%)		
Know partner HIV status				
Yes	139 (92.7)	11 (7.3)	20.9 (10.52,41.85)	16.9 (8.11,35.21)*
No	62 (37.6)	103 (62.4)	1	1
Since they know their status				
≤ 2 years	63 (50.8)	61 (49.2)	1	1
> 2 years	138 (72.3)	53 (27.7)	2.5 (1.57,4.04)	3.2 (1.7,6.29)*
Got pretest counseling				
Yes	177 (66.8)	88 (33.2)	2.1 (1.18,4.01))	2.8 (1.83,4.28)
No	24 (48)	26 (52)	1	1
Thinking that initiating ART important for disclosure				
Yes	171 (67.6)	82 (32.4)	2.2 (1.26,3.9)	1.7 (0.81,3.54)
No	30 (48.4)	32 (51.6)	1	1
Have you had sex in the last 6 months				
Yes	131 (75.3)	43 (24.7)	3.1 (1.91,4.93)	3.2 (1.7,6.19)*
No	70 (49.6)	71 (50.4)	1	1
Seen a person with HIV positive disclose his/her status to the community				
Yes	111 (75.5)	36 (24.5)	2.6 (1.64,4.33)	2.1 (1.08,4.01)*
No	90 (53.6)	78 (46.6)	1	1
Women’s treatment category				
ART	180 (66.7)	90 (33.3)	1	1
Pre ART	21 (46.7)	24 (53.3)	0.4 (0.23,0.82)	0.6 (0.24,1.61)
Use condom				
Yes	97 (83.6)	19 (16.4)	4.1 (2.03,8.47)	1.4 (0.65,2.71)
No	32 (55.2)	26(44.8)	1	1
Prior discussion before test				
Yes	86 (86.9)	13 (13.1)	5.81 (3.068,11.04)	4.87 (2.45, 9.71)*
No	115(53.2)	101(46.8)	1	1

*Significant at p value < 0.05.

## Discussion

The study assessed the magnitude and factors affecting disclosure of HIV positive status to their sexual partners in Mekelle hospital. The overall disclosure rate of HIV status of women to their sexual partner was 63.8%. On the multiple logistic regression, knowledge of sexual partner’s HIV status, receiving pretest counseling, duration since HIV testing, knowing people who disclose their status to the community and having a discussion with their partners prior to HIV testing were the predictor of HIV disclosure.

The entry point for an effective HIV/AIDS care and treatment, including adherence to the ART drug and having a successful PMTCT intervention is disclosure of HIV status to a sexual partner. However, in most of the developing countries, including Ethiopia, women are economically dependent on men, and this might have an effect in expressing their status freely to their sexual partner. Besides, in this study one third women had no discussion with their partner prior to the HIV test and this might have an effect of having a low disclosure rate. Consistent findings were also found in studies conducted in Mettu and Gore town (69%) [5]. However, it is less than other studies conducted in the USA (82%), Kemissie Health Center (93.1%), Hawassa referral hospital (85.7%), California (100%) and Jimma (85%) [7, 12–15].

Being a member of HIV/AIDS association could provide women with information regarding the merit of disclosure as well as for sharing and discussing their problems freely with other individuals so as to help them in acquiring possible solution. This in turn might have an effect in disclosure of HIV status. However, this study report that a large number (71.7%) women were not a member as compared with neighbored countries like Republic of Tanzania (22%) and Rwanda (50.9%) [16, 17].

It is obvious that, as the patients stay longer duration in HIV care services, they are informed about the benefits of HIV/AIDS care treatment, including disclosure, experience sharing with others and got ongoing counseling. These services may encourage the women to disclose their HIV status to their partner. Similar findings were shown in different studies [17–19] - including this study in which disclosure to sexual partners increased from 18% within one month of diagnosis to 42% to six months.

Women had different reasons not to disclose their HIV status or not. Moreover, this study revealed that getting support from a sexual partner, partners help to get medical and ART care and the intention to decrease HIV transmission of HIV/AIDS to their partner were the main reason mentioned by the women to disclose their HIV status. On the other hand, fear of abandonment, having not enough time to discuss with their partner, fear of physical abuse, stigma and discrimination and accusation of infidelity were the main reasons mentioned by the respondents for their non-disclosure. The finding of this study was similar to studies conducted in Mettu and Gore town, Kemissie health center and Hawassa University Referral Hospital [5, 12, 13].

The presence of model person is important to increase people doing the action. In line to this, the study revealed that women who had seen somebody disclosing his/her HIV status to the community were more likely to disclose their HIV status to their partner. This was consistent with a finding of the review paper by the World Health Organization and a study done in Uganda [20, 21].

Knowledge of partner’s HIV status also determined the decision to disclose their HIV status to their partner and this is comparable with the finding of studies conducted in Kemissie Health Center, Hawassa referral hospitals, Jimma University specialized Hospital [12, 13, 15]. HIV positive status of their partner might have helped women to have open communication and freedom to disclose their status without any fear.

Sharing of one’s own ideas with health providers and other clients could develop self confidence to describe their status to their partners and the community. One of the objectives of the counseling during HIV testing to psychologically help women, relief stress, anticipate the consequences and opportunity that could happen regardless of the test result. Hence, women who had pretest counseling had a higher proportion of disclosing their status to their partners. This is also consistent with other studies [17, 20].

This study showed that women who had prior discussions with their partner about HIV and HIV test were more likely to disclose their status than their counterpart. This finding corresponds with studies done in Gore and Metu, Kemissie Health Center and Jimma university specialized Hospital [5, 12, 15]. This might be due to the fact that, communicating with one’s partner prior to HIV testing is a key point in individuals to anticipate a partner’s reaction and would give them an opportunity to raise the issues and disclose their results.

Furthermore, this study revealed that women who considered initiation of ART is important for disclosure were more likely to disclose their HIV status than their complement. This finding was similar to the study done in Tanzania on PLWHA on assessing the impact of initiation of HAART, which showed that 59.5% males and 48.7% females disclosed their HIV test result to a sexual partner after 12 months of initiation of HAART and in Mityana district of Uganda [16, 21]. This could be explained by the fact that initially people on ART receive pre ART counseling, where disclosure is vital to the HIV/AIDS care and support. Secondly, ART was initiated by people who have already developed overt AIDS symptoms and signs that cannot easily be concealed from their partners.

Obtaining a continuous counseling at each contact with health providers and use of different behavior changing techniques might help for patients develop healthy behaviors, including disclosure of HIV sero positive status to sexual partners. Moreover, being women of developing countries in which most of the resources are in the palms of their husband, knowing their sexual partner status is crucial for disclosing their status freely. Different studies [13, 15] agree on the fact that women who knew their sexual partner’s HIV status were more likely to disclose their HIV sero status than their counterparts.

It is obvious that a change in behavior is as a result of learning. Education is a vital to bring about behavioral change within a short period of time. Moreover, having a smooth relationship as well as sharing the same house could help for the women to express her feeling freely as well as makes them to get psychological and social support. However, unlike other studies, educational status, condom usage, having smooth relationships with their partners, living within the same house with their partner and being married were not significantly associated with HIV positive status disclosure. This variation may be attributable to the differences in the sample size and the design. The design computed the sample size based on the proportion of women with respect to HIV disclosure; it didn’t consider the proportion of women needed based on the independent factors such as education.

## Conclusion

The proportion of HIV status disclosure is low. Knowledge of HIV status of partner, receiving pretest counseling, longer time for the HIV testing, know people who disclose their status to the community and having discussion prior to HIV testing could influence disclosure of HIV status of mothers to their partners.

## Abbreviations

AIDS: Acquired Immune Deficiency Syndrome
ART: Anti-Retroviral Therapy
CI: Confidence interval
EDHS: Ethiopia Demographic Health Survey
HAART: Highly Active Anti Retroviral Treatment
HIV: Human Immune-Deficiency Virus
MUCHS: Mekelle University College of Health Science
OR: Odds Ratio
PLWHA: Patient Living with HIV/AIDS
PMTCT: Prevention of Mother to Child Transmission
SPSS: Statically Package for Social Science
UN: United Nation
UNAIDS: United Nation joint program on HIV/AIDS
UNICEF: United Nation Children’s Fund
VCT: Voluntary Counseling and Testing
WHO: World Health Organization.
